# Opportunities for Graduate Study in Special Hospitals

**Published:** 1908-09-05

**Authors:** 


### OPPORTUNITIES FOR GRADUATE STUDY IN SPECIAL HOSPITALS.

### The London Post-graduate Association.

The "London Post-Graduate Association" issues tickets which admit to the clinical practice of a number of the leading general and special hospitals in the metropolis. The fee for a term of three months is £10 10s. Applications should be addressed to the Secretary, London Post-Graduate Association, Examination Hall, Victoria Embankment, W.C.

### Ophthalmic Hospitals.

Ophthalmic hospitals are the Central London Ophthalmic Hospital, Gray's Inn Road, W.C.; the Royal London Ophthalmic Hospital, City Road, E.C.; the Royal Eye Hospital, St. George's Circus, S.E.; and the Royal Westminster Ophthalmic Hospital, King William Street, W.C. In each of these the fees charged are very modest, and full particulars can be obtained on application to the hospital secretary.

### St. John's Hospital for Diseases of the Skin.

Out-patient Department, Leicester Square; In-patient Department, Uxbridge Road, W. This hospital has a well-equipped in-patient department, with 40 beds. It has a School of Dermatology at 49 Leicester Square, which is conducted by the medical staff of the hospital. During the past year the free course of Chesterfield lectures given by Dr. Morgan Dockrell proved a geat success, being well attended by the profession. The next course (free) will commence on Thursday, October 8, at 6 p.m., in the lecture room of the Hospital, Leicester Square. The subject of the opening lecture will be "Cataphoresis in General and Special Medicine." The out-patient department has recently been rebuilt at a cost of £10,000, and contains a spacious laboratory and special electrical department which can be seen in operation every afternoon except Saturday. Clinical demonstrations are given every Monday (Dr. Morgan Dockrell) at 2 p.m.; Tuesday (Dr. Eddowes), at 2 p.m.; Wednesday (Dr. Savill), at 3 p.m., on selected cases; Thursday (Dr. Hargreaves), at 2 p.m.; and Friday (Mr. Dawson), at 2 p.m.

### Queen Charlotte's Lying-in Hospital, Marylebone Road, N.W.

Qualified Medical Pactitioners and Medical Students attending this hospital are permitted to do so for four weeks for a fee of £8 8s. The new Residential College is at No. 5 Cosway Street, N.W. This instruction will include :—(1) Practical instruction in the methods of examination of pregnant women; (2) delivery of women in labour under the direct supervision of a medical officer of the hospital; (3) practical instruction in the treatment of the mother and child during the puerperium, including clinics held four times weekly by the visiting medical staff; (4) instruction in the clinical laboratory of the hospital. The inclusive fee for hospital practice and board and lodging will be £12 12s. per calendar month. 1,701 women were delivered in the hospital last year (1907), of which nearly two-thirds were primiparæ. A large number of the cases were abnormal. Qualified practitioners and medical students will still be admitted under the old regulations, and will be required to attend for a month's course. During this time they will be able to attend the number of cases of labour required by the various examining bodies. The inclusive fee for this course will be £15 15s. as heretofore.

### The Hospital for Women, Soho.

The hospital contains sixty beds. In the out-patient department there were over 4,000 new cases during the past year, the total number of out-patient attendances being 16,338. This large number affords opportunities for examining and studying most of the varieties of diseases peculiar to women. Clinical assistants to the gynæcologists in charge of the out-patient department are appointed for one or more months. They are expected to attend twice weekly in the afternoon. The appointments are open to qualified medical practitioners of either sex. Clinical assistants receive notice of all operations performed within the hospital, and every facility is afforded them in the out-patient department of obtaining experience in diagnosis and treatment and the practical use of instruments. Fee for one month, two guineas, for each subsequent month, two guineas. A certificate is given at the end of a three-months' course. Any further information can be obtained by writing to the Dean at the hospital.

### National Hospital for the Paralysed and Epileptic, Queen Square, W.C.

The out-patient practice of this hospital is open without fee from 2.30 p.m. every day (except Thursday and Saturday), when patients are seen by the physicians for out-patients and the assistant physicians. The in-patient practice is only open to those who take out the hospital course. Three courses of lectures and clinical demonstrations will be given in each year. The out-patient hospital practice and lectures are free, but for the hospital course a fee of two guineas for three months or three guineas for six months is charged. The museum is available for the prosecution of special work under the pathologist, for which a separate fee of two guineas for three months is charged. Apply to the Secretary at the hospital.

CENTRAL LONDON THROAT AND EAR HOSPITAL, GRAY'S INN ROAD, W.C.

Last year 564 in-patients were treated and 9,993 out-patients were seen, involving 46,859 separate attendances. Operation days : In-patients, Tuesday, Wednesday, Thursday, and Friday, at 2 P.M. ; and out-patients, Tuesday, Wednesday, Thursday, and Friday, at 9 A.M. Special courses of practical demonstrations are given weekly on Tuesday and Friday at 4 P.M. during the winter and summer sessions by the members of the staff. They are so arranged that practitioners joining at any time are enabled to complete the group of subjects in a course of six weeks. The fee for this course, with daily attendance at the out-patient department, is three guineas. The fee for general clinical attendance is five guineas for three months and eight guineas for six months. All information can be obtained on application to the Dean, Dr. Wyatt Wingrave, or to Richard Kershaw, Secretary.

HOSPITAL FOR DISEASES OF THE THROAT, GOLDEN SQUARE, LONDON, W.

Clinical instruction in the diagnosis and treatment of disease is given daily in the out-patient department from 2.30 to 5 P.M., on Tuesdays and Fridays from 6.30 to 9 P.M., and on Mondays at 9.30 A.M. Practitioners and medical students are admitted to the practice of the hospital at a fee of five guineas for three months, seven guineas for six months, or ten guineas for perpetual studentship. From amongst the students junior and senior clinical assistants are appointed periodically. Apply to Charles A. Parker, Esq., F.R.C.S., Hon. Med. Sec.

